# Monocytes Play an IL-12-Dependent Crucial Role in Driving Cord Blood NK Cells to Produce IFN-g in Response to *Trypanosoma cruzi*

**DOI:** 10.1371/journal.pntd.0002291

**Published:** 2013-06-20

**Authors:** Aline Guilmot, Julie Bosse, Yves Carlier, Carine Truyens

**Affiliations:** Laboratoire de Parasitologie, Faculté de Médecine, Université Libre de Bruxelles (U.L.B.), Brussels, Belgium; Federal University of São Paulo, Brazil

## Abstract

We previously reported that foetuses congenitally infected with *Trypanosoma cruzi*, the agent of Chagas disease, mount an adult-like parasite-specific CD8^+^ T-cell response, producing IFN-g, and present an altered NK cell phenotype, possibly reflecting a post-activation state supported by the ability of the parasite to trigger IFN-g synthesis by NK cells *in vitro*. We here extended our knowledge on NK cell activation by the parasite. We compared the ability of *T. cruzi* to activate cord blood and adult NK cells from healthy individuals. Twenty-four hours co-culture of cord blood mononuclear cells with *T. cruzi* trypomastigotes and IL-15 induced high accumulation of IFN-g transcripts and IFN-g release. TNF-a, but not IL-10, was also produced. This was associated with up-regulation of CD69 and CD54, and down-regulation of CD62L on NK cells. The CD56^bright^ NK cell subset was the major IFN-g responding subset (up to 70% IFN-g-positive cells), while CD56^dim^ NK cells produced IFN-g to a lesser extent. The response points to a synergy between parasites and IL-15. The neonatal response, observed in all newborns, remained however slightly inferior to that of adults. Activation of IL-15-sensitized cord blood NK cells by the parasite required contacts with live/intact parasites. In addition, it depended on the engagement of TLR-2 and 4 and involved IL-12 and cross-talk with monocytes but not with myeloid dendritic cells, as shown by the use of neutralizing antibodies and cell depletion. This work highlights the ability of *T. cruzi* to trigger a robust IFN-g response by IL-15-sensitized human neonatal NK cells and the important role of monocytes in it, which might perhaps partially compensate for the neonatal defects of DCs. It suggests that monocyte- and IL-12- dependent IFN-g release by NK cells is a potentially important innate immune response pathway allowing *T. cruzi* to favour a type 1 immune response in neonates.

## Author Summary

IFN-g release by NK cells is essential in early control of infections with intracellular pathogens by driving protective type 1 immune response. NK cell activation requires integration of signals delivered by cytokines, dendritic cells, monocytes/macrophages and/or pathogens. Little information is available about this topic in neonates, known to be deficient in mounting type 1 immune response. We show that *Trypanosoma cruzi*, the protozoa agent of Chagas disease, rapidly and strongly up-regulates the production of IFN-g by IL-15-primed cord blood NK cells to a level close to that produced by adult NK cells. This neonatal NK cell response was dependent on cross-talk with monocytes and engagement of TLR2 and TLR4 by the parasite. Importantly, IL-12 synthesis by monocytes, but not by dendritic cells, was central in driving NK cell IFN-g release. This study suggests that monocytes may compensate for the known defects of neonatal DCs to produce IL-12. This innate pathway may allow a pathogen to circumvent the defect to mount type 1 immune response in early life. This observation may be relevant *in vivo* in *T. cruzi* congenital infection, since such newborns have previously been shown to mount an adult like type 1 immune response.

## Introduction

Chagas disease, caused by the protozoa *Trypanosoma cruzi*, is a major cause of cardiac failure in Latin America where it infects 8–10 million people [1]. Parasites are mainly transmitted by blood-sucking vector bugs, transfusion of infected blood, or from mothers to their foetuses. Our previous studies in infants from chagasic mothers showed *T. cruzi* being a potent activator of both innate and adaptive immune responses in early life. Indeed, neonates congenitally-infected with *T. cruzi* mount a mature parasite-specific CD8^+^ T lymphocyte response producing IFN-g [2], whereas uninfected infants from *T. cruzi*-infected mothers (probably by receiving circulating parasite molecules from their mothers) display a pro-inflammatory environment associated with activated monocytes [3]. In addition, both congenitally infected and uninfected infants from chagasic mothers develop boosted type 1 immune responses to vaccines routinely administered in early life [4]. These data point out the ability of *T. cruzi* to overcome the immune deficiency associated with early life [5], [6].

NK cells mediate protection against pathogens through secretion of IFN-g that activates phagocytes and shape Th1-dependent adaptive immune response, as well as through destruction of infected cells by their natural cytotoxic properties. These functions are associated with distinct human NK cell sub-populations identified by their differential expression of CD56 and CD16: the CD56^dim^CD16^+^ subset is preferentially cytotoxic, while the CD56^bright^CD16^−/low^ subset is specialized in cytokine production. NK cells express a repertoire of inhibiting and activating receptors recognizing self-ligands or microbial molecules expressed on infected and tumour cells. The balance between signals delivered by these receptors tightly regulates their responses [7], [8]. Cytokines (IL-2, IL-15, IL-12, IL-18 and type 1 IFNs) and contact-dependent signals provided by dendritic cells (DCs), monocytes/macrophages and CD4^+^ T cells also contribute to NK cell activation [9]–[12]. Though neonatal NK cells display some functional defects [13], their intrinsic ability to produce IFN-g seems comparable to adults [14]–[16]. Nevertheless, the reduced ability of neonatal mononuclear cells to produce NK cell-activating cytokines likely hinders their IFN-g response [14]. Information on in vivo NK cell responses to pathogens in early life is scarce owing to the difficulty to perform such studies [13].

By investigating the functional properties of NK cells from *T. cruzi*-congenitally infected newborns, we previously showed they display a defective ability to produce IFN-g in response to cytokines and a reduced cytotoxic capacity at birth. These alterations might however reflect a down-regulated state of NK cells after activation having occurred *in utero* when the parasite was transmitted. This possibility is sustained by our observation that *T. cruzi* was able to trigger in vitro IFN-g synthesis by cord blood NK cells [13], [17]. This is also in line with results reported by Sathler-Avelar et al suggesting that NK cells are activated during acute *T. cruzi* infection in infants [18]. We here confirm the ability of *T. cruzi* to induce IFN-g production by blood NK cells from a large cohort of healthy newborns, compared its effect to that on adult cells, and investigated the mechanism of activation of neonatal NK cells.

## Materials and Methods

### Ethics statement

The ethical committee of U.L.B. has approved this study (protocol # P2011/254), and we obtained informed written consent from volunteers and mothers.

### Patients and blood collection

Umbilical cord blood (CB) samples from full-term healthy newborns, born from healthy mothers, were harvested in heparinized tubes at the maternity ward of the Erasmus Hospital (Brussels). Adult peripheral blood (PB) samples were obtained from healthy volunteers who have been tested to be negative in *T. cruzi* serology. Blood samples were used within 8 hours of collection.

### *T. cruzi* parasites

Live *T. cruzi* trypomastigotes and lysate (Tulahuen strain, genotype VI [19]) were obtained as previously described [3]. Absence of *Mycoplasma* was verified by PCR (Lucron Bioproducts).

### Cell sample isolation and culture

CB and PB mononuclear cells (MC) were isolated by Nycoprep density gradient centrifugation. Their viability was ≥98% as determined by trypan blue exclusion staining. Cells (5×10^5^) were distributed in polypropylene tubes in RPMI 1640 (1 mL) containing 10% heat-inactivated fetal calf serum, penicillin G and streptomycin. They were incubated with recombinant human interleukin-15 (20 ng/mL) (R&D Systems) and/or live or lysed *T. cruzi* trypomastigotes in a 1∶1 parasite-to-cell ratio for 24 h at 37°C (5% CO_2_). Cells incubated in medium alone were used as control.

In cultures designed to detect intracellular IFN-g, brefeldin A (10 µg/mL, Sigma-Aldrich) was added for the last 4 hours of culture. For IL-12p70 blocking experiments, CBMC were incubated with anti-human IL-12 monoclonal antibody that does not cross-react with IL-23 (clone 24910, R&D Systems), or control unrelated IgG (Sigma-Aldrich). Magnetically depletion of CD1c^+^ myeloid (m)DCs or CD14^+^ monocytes were performed using anti-CD1c or -CD14 microbeads, LD columns and MidiMACS equipment (all from Miltenyi Biotec) as described by the manufacturer. This led to depletion of 97.6±1.2% mDCs and 93.8±1.8% monocytes. mDCs or monocytes-depleted CBMC and reconstituted CBMC (purified mDC or monocyte fraction added in CBMC depleted fraction) were cultured as described above.

To determine if cell-cell contacts were involved in activation of NK cells, an insert with a semi-permeable membrane (pore size of 0.4 µM, Greiner Bio-One) was used. Monocyte-depleted CBMC were cultured in the lower part of the transwell, purified autologous monocytes were added into the upper well, while parasites were added at both sides. Controls used reconstituted CBMC in both sides. Transwell experiments were also performed to determine the need for contact between parasites (upper side of the membrane) and CBMC (lower side).

After stimulation, cell cultures were centrifuged at 750 g for 5 min at room temperature and supernatants were kept at −70°C for cytokine assays. Cells were further processed for flow cytometry analyses or quantitative RT-PCR. Viability of NK cells (≥98%) and monocytes (≥92%) was verified by flow cytometry using the LIVE/DEAD Viability Assays (Invitrogen) and was not modified whatever the conditions of stimulation.

### Cytokine assays

IFN-g, TNF-a and IL-10 levels in culture supernatants were detected by ELISA using antibody pairs and standards from Biosource (Invitrogen). IL-18 was detected using the anti-IL-18 clones 125-2H and 159-12B (R&D Systems) for coating and detection, whereas IL-12p70 was detected using READY-SET-GO! IL-12p70 kit (eBioscience). Assays were performed in duplicate following the manufacturer's instructions. Detection limits were 2 pg/mL for all cytokines.

### Flow cytometry analyzes

Extracellular and intracellular stainings were performed as previously described [2], using the following mAb and their matched control isotypes in various combinations : anti-human (h) CD3-peridinin chlorophyll protein (PercP), anti-hCD11c-allophycocyanin (APC), anti-CD14-fluorescein isothiocyanate (FITC), anti-hCD16-phycoerythrin (PE), anti-hCD19-PE, anti-hCD34-FITC, anti-hCD54-PE, anti-hCD62L-PE, anti-hCD69-FITC, anti-hCD123-PE, anti-hIFN-g-FITC, anti-hIFN-g- PE, Lin1-FITC (BD Biosciences), anti-hCD56-APC, anti-hHLA-DR-PerCP, anti-hIL-12p35-PE (Miltenyi Biotec), anti-TLR-2-PE and anti-TLR-4-PE (e-Biosciences). Data acquisition was stopped when 1000 events was reached for the CD56^bright^ NK cell subset or 5000 events for the CD14^+^ monocytes. Data acquisition and analyzes were performed using a four-colour FACSCalibur flow cytometer and CELLQuest 6.0 software (BD). NK cell analyzes were made on CD56^bright^CD16^−/low^ and CD56^dim^CD16^+^ cells, targeted in CD3^−^ cells present in a large lymphocyte gate determined in the SSC-FSC plot. The relative proportions of NK cell subsets were concordant with previous reports [13]. Proportions of the CD56^bright^ subset were similar in cord and adult samples (13.2±1.3% and 10.1±1.6% respectively) and slightly increased (by 1.1–1.3 fold) in the presence of parasites and/or IL-15. Monocyte analyzes were made on CD14^+^ cells. Limits for the quadrant markers were set on negative populations and isotype controls. Results are presented as percentages of cells expressing the analyzed marker or as geometric mean fluorescence intensity (MFI) of the total cell population. Depletion of monocytes and mDCs was verified by analyzing population of untouched and depleted CBMC (identifying monocytes as CD14^+^ cells and mDCs as CD11c^+^CD123^−^HLA-DR^+^Lin1^−^CD34^−^ cells).

### Quantitative reverse transcription polymerase chain reaction (qRT-PCR)

Total RNA was isolated using High Pure RNA Isolation Kit (Roche Applied Science) as recommended by the manufacturer. The amount and purity of RNA were determined by spectrophotometry. Four hundred ng of each sample of RNA have been used for subsequent RT-PCR process on Mastercycler ep gradient (Eppendorf) using the Transcriptor First Strand cDNA Synthesis Kit (Roche) with oligo-dT primers following the manufacturer's instructions. Reverse-transcripted RNA samples were half-diluted and processed by real-time PCR on the Lightcycler 480 (Roche) using SYBR Green Supermix (Quanta Biosciences/VWR) and the following primers (InVitrogen): IFN-g (5′-ACTGACTTGAATGTCCAACGCA-3′ and 5′-ATCTGACTCCTTTTTCGCTTCC-3′ [20]), IL-12p35 (5′-TTCACCACTCCCAAAACCTGC-3′ and 5′-GAGGCCAGGCAACTCCCATTAG-3′ [21]), IL-18 (5′-CAGACCTTCCAGATCGCTTC-3′ and 5′-GGGTGCATTATCTCTACAGTCAGAA-3′ [22]) and GAPDH (5′-AACAGCCTCAAGATCATCAGC-3′ and 5′-GGATGATGTTCTGGAGAGCC-3′ [23]). PCR protocol consisted in a denaturation phase at 95°C for 5′ and 50 cycles of amplification [95°C 3″, 60°C 1′]. Fluorescence emission was measured at the end of the elongation step. The cycle number at which fluorescence emission crossed the determined threshold value was determined. Melting curve analysis was used to assess the specificity of the assay. Fold changes were calculated using the 2^−ΔΔCT^ method with GAPDH as house-keeping gene and unstimulated cells cultured for the same time as control. Each sample was tested in duplicate.

### Expression of results and statistical analysis

Data are expressed as means ± SEM or Box and Whisker plots (showing medians, quartiles and minimum and maximum values). Differences between unstimulated and stimulated cells were tested for significance using Wilcoxon matched-paired test. Comparisons between neonates and adults were performed using Mann Whitney *U* test. Statistical significance was accepted if P<0.05. Statistical analyzes were performed with GraphPad Prism 5.02.

## Results

### *T. cruzi* induces accumulation of IFN-g transcripts and release of IFN-g by cord blood cells

**Figure 1:**
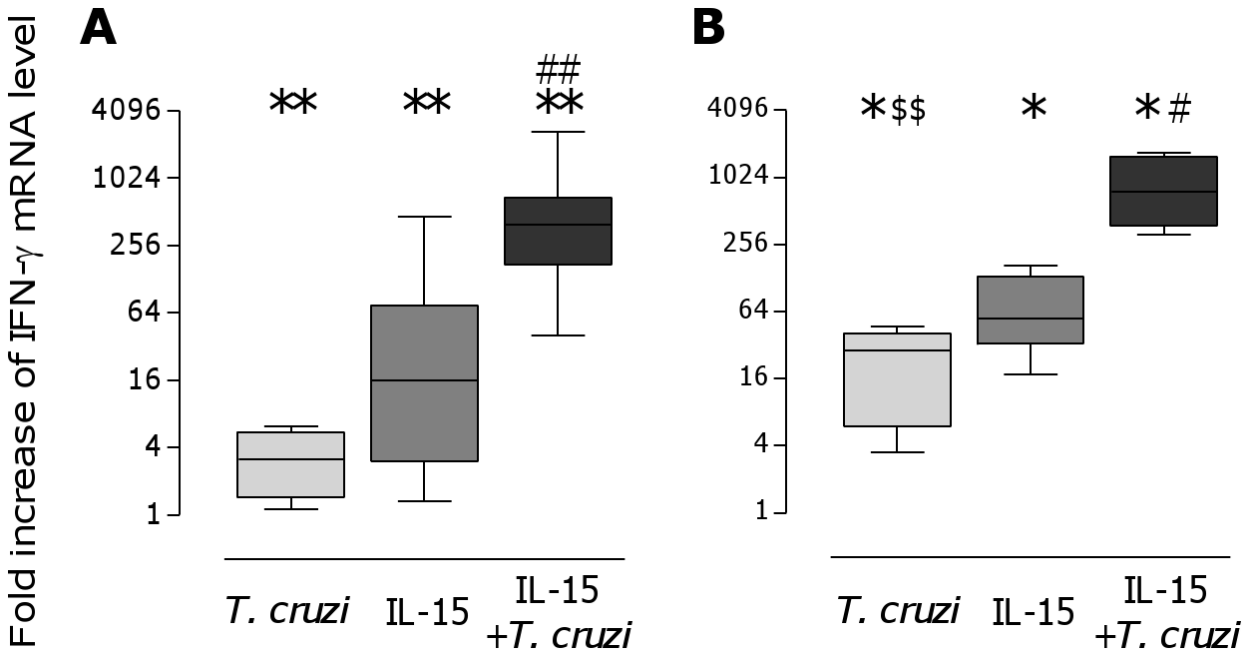
IFN-g mRNA accumulation in cord and adult blood mononuclear cells after IL-15 and *T. cruzi* incubation. Fold increase of IFN-g mRNA levels after 24 h incubation of cord blood mononuclear cells (CBMC - A) or adult peripheral blood mononuclear cells (PBMC - B) in the presence or not of *T. cruzi* live trypomastigotes (parasite∶cell ratio 1∶1) and/or IL-15 (20 ng/mL). Transcript levels are shown as box and whiskers of fold increases as compared to unstimulated cells (n = 9 cord blood and 6 adults blood samples). *: p<0.05, **: p<0.005 as compared with unstimulated cells, #: p<0.05, ##: p<0.005 as compared with the effect of IL-15 alone (Wilcoxon paired test), $$: p<0.005 as compared to cord blood cells Mann-Whitney U test.

CBMC co-cultured for 24 h with *T. cruzi* live trypomastigotes (ratio parasite to cell of 1) associated with IL-15 (20 ng/mL) produced large amounts of IFN-g, low levels of TNF-a and no IL-10 in response to *T. cruzi* associated with IL-15, whereas parasites or IL-15 alone were markedly less effective, suggesting they synergize to trigger the release of these cytokines (**Table 1**). The synergy between parasites and IL-15 was also noticeable at the level of IFN-g transcript accumulation (**Figure 1A**). A similar profile of response was observed in adult PBMC, though IFN-g transcript level and cytokine production in response to parasites and IL-15 were around two fold higher than in CB cells (**Table 1** **and** **Figure 1B**).

**Table 1:** Production of cytokines by cord and adult blood cells in response to *T. cruzi* and/or IL-15.

Sample	Cytokine pg/mL^a^	NS^b^	*T. cruzi* b	IL-15^b^	IL-15+*T. cruzi* b
**Cord blood**	IFN-g	2,00 (2,0 - 2,0)	2,00 (2,0 - 3,4)	27,55 (2,0 - 358,6) *	925,2 (122,3 - 1924) * #
	TNF-a	2,00 (2,0 - 5,6)	11,60 (2,0 - 104,3) *	11,65 (2,0 - 126,5) *	209,0 (56,6 - 1495) * #
	IL-10	2,00 (2,0 - 2,0)	2,00 (2,0 - 2,0)	2,00 (2,0 - 2,0)	2,00 (2,0 - 2,1)
	IL-12p70	2,00 (2,0 - 2,0)	2,00 (2,0 - 2,0)	2,00 (2,0 - 2,0)	2,00 (2,0 - 3,3)
	IL-18	2,00 (2,0 - 2,3)	2,00 (2,0 - 2,0)	2,00 (2,0 - 2,0)	2,00 (2,0 - 2,0)
**Adult blood**	IFN-g	2,00 (2,0 - 2,0)	2,80 (2,0 - 42,3)	150,6 (6,2 - 788,4) *	2396 (521,8 - 2830) * # $
	TNF-a	2,00 (2,0 - 31,8)	50,05 (2,0 - 185,2) *	48,80 (2,0 - 504,9) *	966,5 (448,5 - 1972) * # $
	IL-10	2,00 (2,0 - 2,0)	2,00 (2,0 - 2,0)	2,00 (2,0 - 2,0)	2,55 (2,0 - 5,0)
	IL-12p70	2,00 (2,0 - 2,0)	2,00 (2,0 - 2,0)	2,00 (2,0 - 2,0)	2,85 (2,0 - 8,4) * # $
	IL-18	2,00 (2,0 - 7,2)	2,00 (2,0 - 7,7)	2,00 (2,0 - 12,0)	2,00 (2,0 - 10,4)

a Results are shown as medians (min-max values) of 8 cord blood samples and 6 adult blood samples.

b Cord or adult mononuclear cells were culture for 24 h in the presence or not of *T. cruzi* live trypomastigotes (parasite to cell ratio 1∶1) and/or IL-15 20 ng/mL.

* p<0,05 as compared with unstimulated cells (NS), Wilcoxon paired test.

# p<0,05 as compared with the effect of IL-15 alone, Wilcoxon paired test.

$ p<0,05 as compared with cord blood cell response, Mann-Whitney U test.

### CD56^bright^ NK cells are the main IFN-g responding cells to *T. cruzi* associated with IL-15

**Figure 2:**
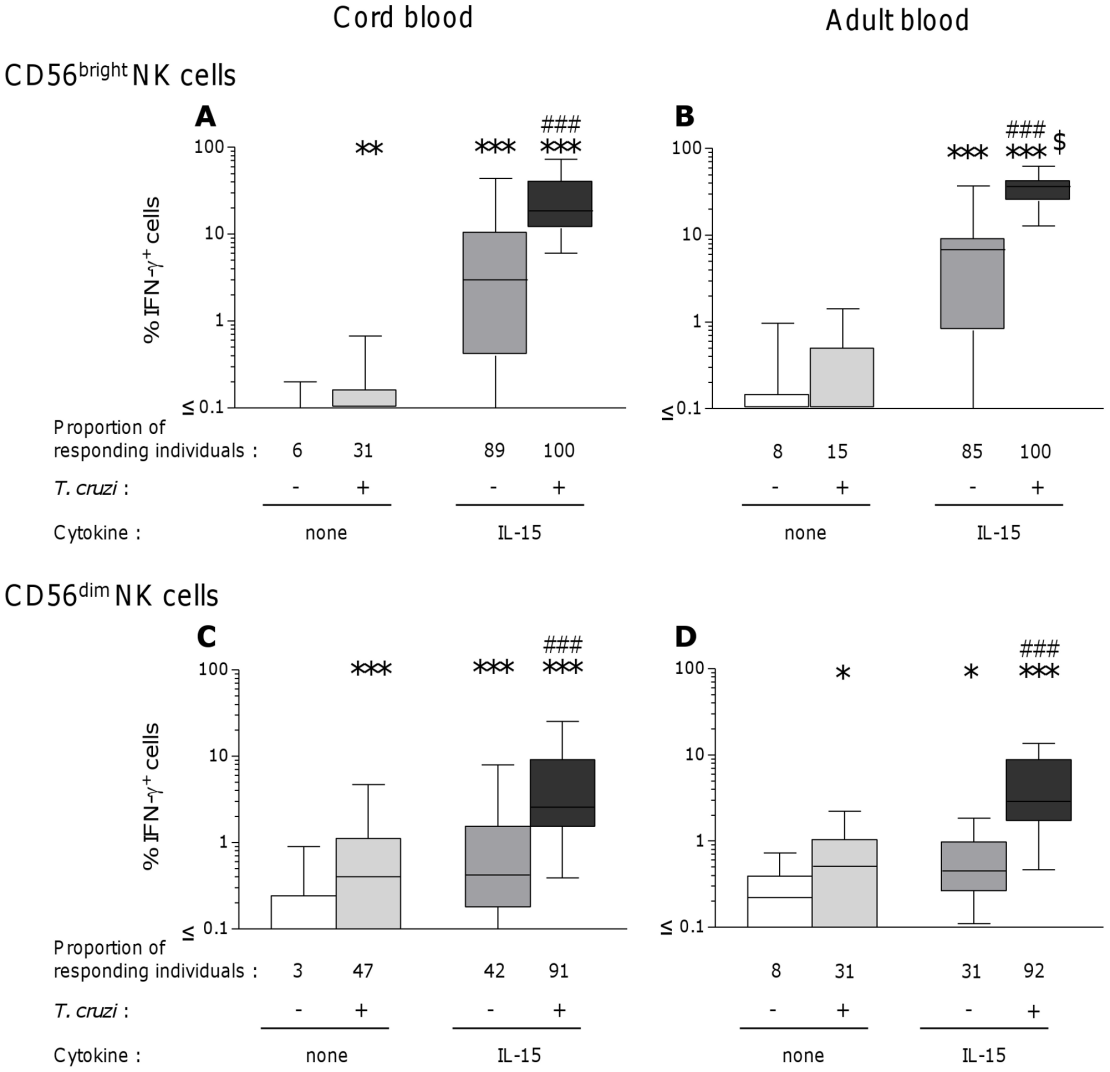
Intracellular detection of IFN-g in CD56^bright^ and CD56^dim^ NK cells after IL-15 and *T. cruzi* incubation. Proportion of blood CD56^bright^ (AB) and CD56^dim^ (BD) NK cells from neonates (A and C) and adults (B and D), expressing IFN-g after 24 h incubation with *T. cruzi* live trypomastigotes (ratio 1 parasite per cell) in the presence or not of rhIL-15 (20 ng/mL). Results are shown as Box-and-Whisker plots (n = 36 cord and 13 adult blood samples). Proportions of responding individuals correspond to percentages of samples presenting a proportion of IFN-g positive cells above the cut-off, calculated as the mean+2SD of the % of IFN-g+ cells observed without stimulation. *: p<0.05, **: p<0.005, ***: p<0.0005 as compared with unstimulated cells, ###: p<0.0005 as compared with the effect of IL-15 alone (Wilcoxon paired test), $: p<0.05 as compared to cord blood cells (Mann-Whitney U test).

Flow cytometry analysis of IFN-g producing cells in response to *T. cruzi* and IL-15 allowed to identify NK cells as major responding ones, since less than 0.15 (cord) to 0.6 (adult) % of T cells and no other cells contained IFN-g in the tested conditions (data not shown). Parasites or IL-15 alone weakly triggered IFN-g synthesis by a low proportion of CD56^bright^ NK cells in some CB samples (**Figure 2A**). Strikingly, both signals strongly synergized to boost the IFN-g response by around 6 times, leading to production of IFN-g by meanly 20% of CD56^bright^ NK cells (up to 70% in some individuals). It is to notice that 100% of newborns respond to parasites and IL-15. In adult cells, *T. cruzi* alone did not significantly trigger IFN-g production and the proportion of CD56^bright^ NK cells producing IFN-g in response to IL-15 alone was comparable to that found in cord blood cells (medians 7% vs. 3%, p>0.05). Combination of parasites and IL-15 similarly synergized to increase IFN-g production by adult CD56^bright^ NK cells (**Figure 2B**). Yet, the mean proportion of CD56^bright^ NK cells producing IFN-g after such activation was higher in adult than in cord blood cells (**Figures 2B vs. 2A**, 36 vs. 20%, p = 0.017). CD56^dim^ CB and PB NK cells also produced IFN-g (**Figures 2CD**) though, as expected from the literature [7], [24], [25], the proportion of IFN-g-producing cells remained largely inferior to that observed in CD56^bright^ NK cells.

These results indicate that *T. cruzi* synergizes with IL-15 in triggering IFN-g production by neonatal and adult CD56^bright^ NK cells, and that, despite the response of CB cells was inferior to that of adult cells, IFN-g production can be obtained in all newborns.

### IFN-g synthesis by cord blood CD56^bright^ NK cells is associated with surface expression of activation markers

**Figure 3:**
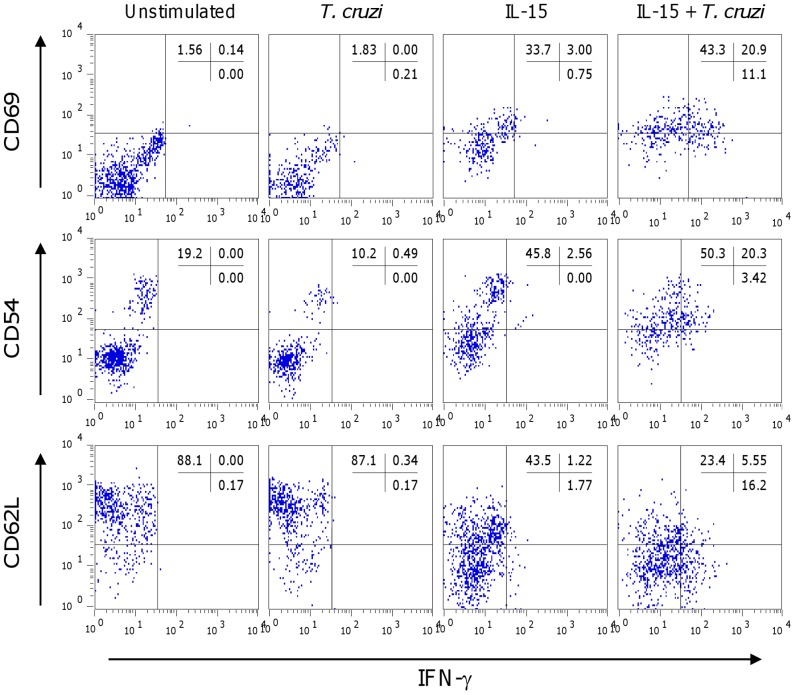
Co-expression of IFN-g and activation markers in neonatal CD56^bright^ NK cells. Representative dot-plots showing co-expression of IFN-g and CD69, CD54 or CD62L in NK CD56^bright^ cells after 24 h stimulation of CBMC in the presence or not of *T. cruzi* live trypomastigotes at a parasite∶cell ratio of 1∶1 and/or IL-15 (20 ng/mL). Data from one representative experiment from 5 giving similar results.

After 24 h of stimulation, IL-15 strongly induced the expression of CD69 and CD54 on around 30% of both cord and adult blood NK cell subsets and down-regulated dramatically CD62L expression, indicating their activation. *T. cruzi* used alone barely activated NK cells, only weakly inducing CD69 expression. Parasites enhanced by 1.5 to 2 fold the IL-15 effect for all the activation markers. The CD56^dim^ NK cells were also activated by IL-15 and further by the combination of IL-15 and parasites. Expression of activation markers by both NK cell subsets from adult blood in response to parasites and/or IL-15 was very comparable to that of cord blood NK cells (**Table 2**). Most IFN-g positive CD56^bright^ NK cells co-expressed CD69 and CD54 and had down-regulated CD62L (**Figure 3**).

**Table 2:** Expression of activation markers on NK cells from cord and adult blood in response to *T. cruzi* and/or IL-15.

NK subset	Parameter^a^	Marker	NS^b^	*T. cruzi* b	IL-15^b^	IL-15+*T. cruzi* b
**Cord blood**						
**CD56^bright^**	% positive cells	CD69	0,81±0,38	1,95±0,64 *	37,58±4,86 *	54,25±5,52 * #
		CD54	8,28±2,59	6,36±1,87	33,89±6,33 *	56,74±5,62 * #
		CD62L	91,27±1,89	90,49±1,75	22,79±4,02 *	15,73±4,05 * #
	MFI	CD69	3,35±0,31	3,53±0,49	27,14±6,49 *	42,31±12,60 * #
		CD54	14,85±1,56	12,55±0,79	41,68±5,51 *	63,05±5,75 * #
		CD62L	204,7±7,47	184,85±7,71	16,23±3,33 *	11,88±2,49 * #
**CD56^dim^**	% positive cells	CD69	2,26±1,06	2,80±1,08 *	31,24±4,74 *	48,04±4,07 * #
		CD54	13,63±2,51	9,64±2,02	22,16±3,40 *	30,98±4,87 *
		CD62L	32,70±4,98	28,57±4,51	12,72±2,94 *	8,34±2,60 * #
	MFI	CD69	3,81±0,39	3,87±0,27	16,48±2,51 *	27,94±5,12 * #
		CD54	9,37±0,73	8,79±0,94	20,02±2,94 *	26,60±1,14 *
		CD62L	16,72±2,54	14,34±2,29	7,50±1,36 *	6,46±1,28 *
**Adult blood**						
**CD56^bright^**	% positive cells	CD69	0,78±0,25	2,30±1,01 *	28,47±3,94 *	58,60±7,32 * #
		CD54	7,39±2,65	7,74±2,47	36,89±4,56 *	69,99±4,01 * #
		CD62L	88,93±3,40	82,38±6,65	32,67±4,04 *	18,84±3,03 * #
	MFI	CD69	4,10±0,23	4,18±0,27	21,87±1,96 *	40,43±5,75 * #
		CD54	14,41±2,64	13,45±1,67	38,94±8,37 *	63,64±6,93 * #
		CD62L	210,77±11,75	195,93±37,16	24,34±2,71 *	13,97±1,76 * #
**CD56^dim^**	% positive cells	CD69	1,79±0,71	3,57±1,37 *	30,80±3,40 *	54,95±6,81 * #
		CD54	20,19±5,72	17,25±6,51	29,24±5,90 *	40,13±4,52
		CD62L	33,86±4,33	30,76±5,41	17,84±5,04 *	9,43±2,52 * #
	MFI	CD69	5,19±0,55	5,13±0,50	21,72±2,36 *	37,48±6,12 * #
		CD54	14,15±3,13	11,44±1,97	30,24±4,90 *	34,40±2,98 *
		CD62L	20,00±1,51	18,56±3,87	9,56±0,50 *	7,41±0,39 * #

a Results are shown as mean ± SEM of 5 to10 cord blood samples and 5 to 8 adult blood samples.

b Cord or adult mononuclear cells were culture for 24 h in the presence or not of *T. cruzi* live trypomastigotes (parasite to cell ratio 1∶1) and/or IL-15 20 ng/mL.

* p<0,05 as compared with unstimulated cells (NS), Wilcoxon paired test.

# p<0,05 as compared with the effect of IL-15 alone, Wilcoxon paired test.

### IL-12 plays a major role in IFN-g production in response to *T. cruzi* and IL-15

**Figure 4:**
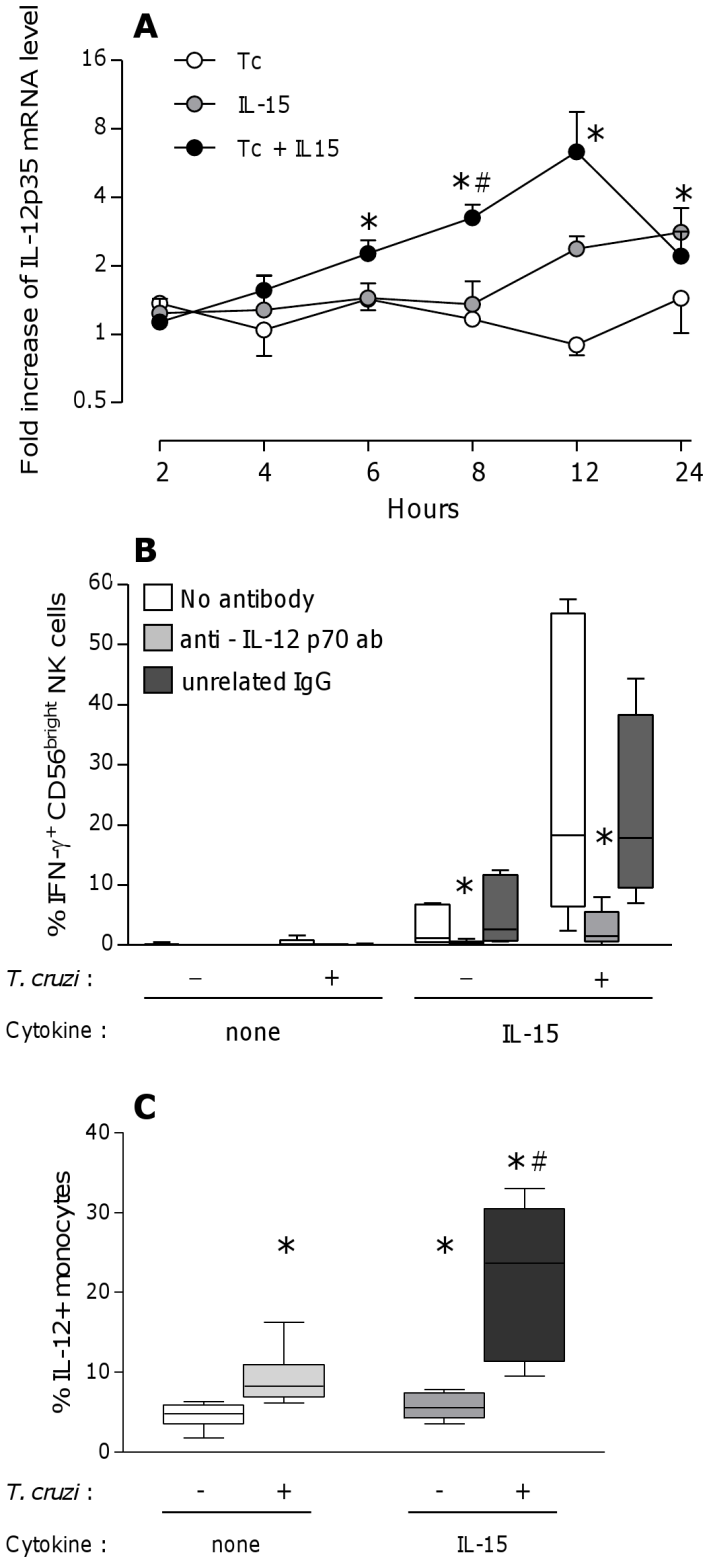
Role of IL-12p70 in IL-15 and *T. cruzi* driven IFN-g production by NK cells. A. Levels of IL-12p35 transcripts in cord blood cells at different time points during culture with *T. cruzi* (ratio 1 parasite per cell) and/or IL-15 (20 ng/mL). Results are shown as mean ± SEM of fold increases as compared to unstimulated cells at same time point (n = 5). *: p<0.05 as compared with unstimulated cells, #: p<0.05 as compared with the effect of IL-15 alone at same time (Wilcoxon paired test). B. Proportion of cord blood CD56^bright^ NK cells expressing IFN-g after 24 h incubation of CBMC with *T. cruzi* (parasite∶cell ratio 1∶1) and/or IL-15 (20 ng/mL) in presence or absence of anti-IL-12p70 blocking antibodies or unrelated IgG. Results are shown as Box-and-Whisker plots (n = 5). *: p<0.05 as compared to CBMC cultured without Ab or with unrelated IgG (Wilcoxon pared test). C. Proportion of cord blood CD14^+^ monocytes expressing IL-12 after 8 h incubation of CBMC with *T. cruzi* (parasite∶cell ratio 1∶1) and/or IL-15 (20 ng/mL). Results are shown as Box-and-Whisker plots (n = 5). *: p<0.05 as compared with unstimulated cells, #: p<0.05 as compared with the effect of IL-15 alone (Wilcoxon pared test).

Since IL-12 and IL-18 are important cytokines for NK cell activation [9], [26] and are induced during *T. cruzi* infection [27], [28], we sought for their potential involvement in activation of CB NK cells. We did not detect significant levels of IL-12 or IL-18 in supernatants of CBMC cultured with *T. cruzi* and IL-15, neither at 24 h (**Table 1**) nor at different time points between 2 h and 24 h of culture (data not shown). On another hand, IL-12p35 (**Figure 4A**) but not IL-18 (data not shown) transcripts accumulated in CB cells. Indeed, IL-12p35 mRNA levels increased by 3 times after 12–24 h of IL-15 stimulation and earlier by 6 times when parasites were added. In line with this, the use of neutralizing anti-IL-12p70 mAb almost totally inhibited IFN-g synthesis by CD56^bright^ NK cells while unrelated control IgG had no effect (**Figure 4B**).

### Activation of NK cells by *T. cruzi* involves contacts with monocytes and intact parasites as well as TLR2 and TLR4 engagements

**Figure 5:**
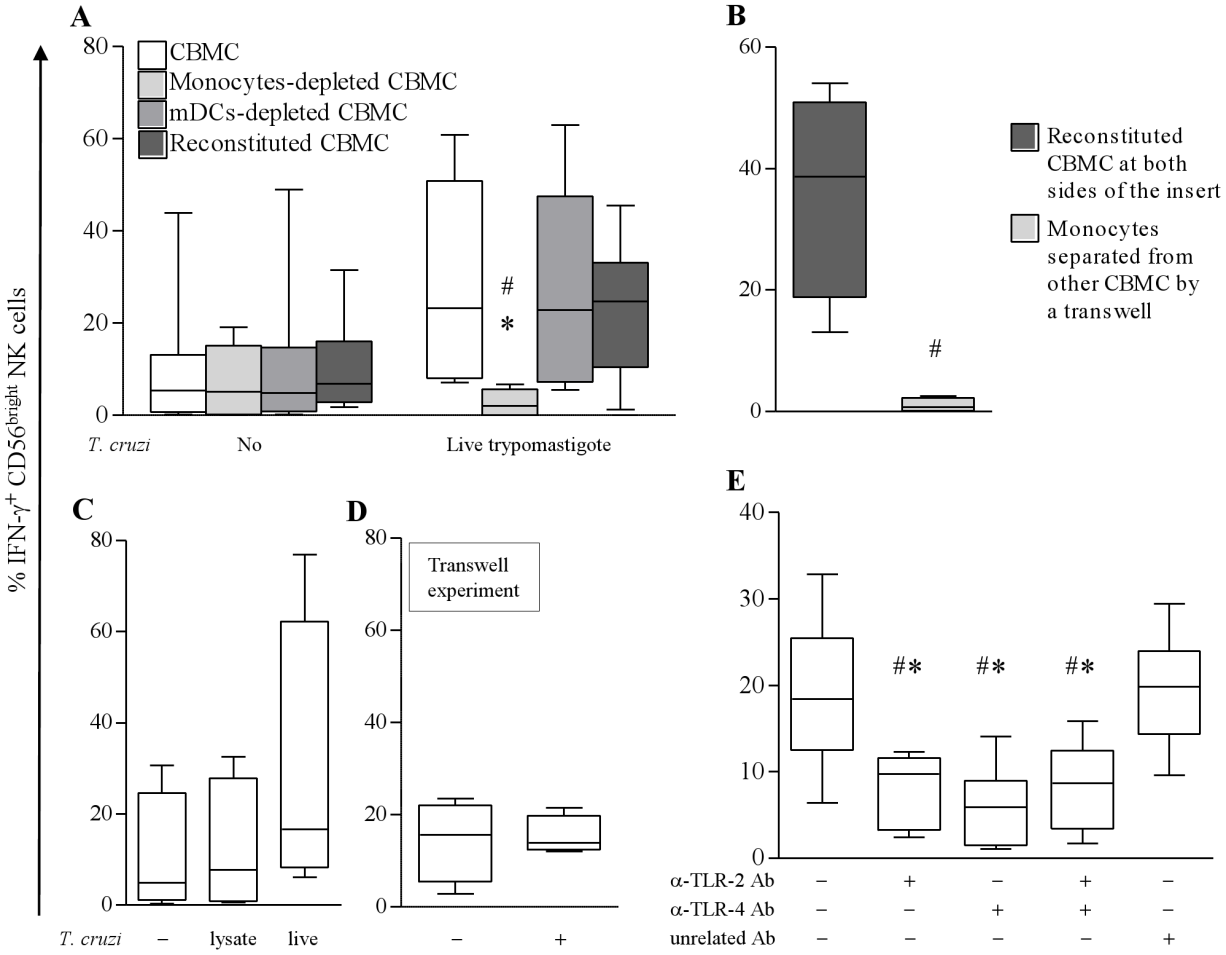
Role of myeloid cells and TLRs in IL-15 and *T. cruzi* driven IFN-g production by NK cells. Proportion of cord blood CD56^bright^ NK cells expressing IFN-g after 24 h incubation of CBMC, monocyte- or mDCs-depleted CBMC or reconstituted CBMC cells (depleted CBMC+purified cells) with or without *T. cruzi* live trypomastigotes at 1∶1 parasite∶cell ratio in presence of IL-15 (20 ng/mL - A). For further investigation (B, C, D), we used only the IL-15+*T. cruzi* 1∶1 condition. To investigate the need for contact between monocytes and other CBMC in activation of NK cells, purified monocytes were cultured at the upper side of a transwell insert while monocyte-depleted CBMC were at the bottom. As control, reconstituted CBMC were cultured at both sides. Parasites were added at both sides (B). To investigate the requirement of live parasites, CBMC were cultured in the presence of live or lysed trypomastigotes (C). To investigate the requirement of contact between CBMC and parasites, CBMC were cultured at the bottom of a transwell insert in the presence or not of parasites at the bottom (D). To investigate the requirement of TLRs, we added anti-TLR-2 and/or anti-TLR-4 blocking Ab or unrelated IgG during culture (E). Results are shown as Box-and-Whisker plots (n = 5–6). * or #: p<0.05 as compared with total CBMC or reconstituted or unrelated IgG treated CBMC respectively (Wilcoxon paired test).

Amongst blood MC, monocytes and mDCs are susceptible to produce IL-12 [26]. We thus looked at the production of IL-12 by these two populations and studied the effect of depleting these cell types on CB NK cell response. After 8 h of stimulation with IL-15 and *T. cruzi*, meanly 25% of monocytes contained IL-12 (**Figure 4C**) while we did not find any IL-12 in mDCs at this timing (data not shown). According with this, depletion of mDCs did not affect IFN-g production by NK cells while monocyte depletion led to a drastic decrease in response to parasites and IL-15 (**Figure 5A**). This decrease was not observed with control reconstituted cells, indicating that the absence of IFN-g synthesis after monocyte depletion was not due to alteration of cells that might have occurred during the depletion procedure.

Cross-talk between NK cells and other cells may involve surface interactions or soluble factors like cytokines [9], [29]. To investigate if NK cell activation was dependent on contact with monocytes, we separated monocytes from monocyte-depleted CBMC by a semi-permeable membrane. Such separation totally abrogated NK cell activation by parasites and IL-15 (**Figure 5B**). On the other hand, trypomastigote lysate or trypomastigotes separated from CBMC by a semi-permeable membrane did not induce synergistic production of IFN-g by IL-15-primed NK cells (**Figure 5CD**).

TLR2 and TLR4 are known to recognize *T. cruzi* PAMPs [30]. We found TLR2 and TLR4 expression on 98.9±0.3% and 76.0±6.8% of CB unstimulated monocytes and 7.6±2.1% and 6.4±1.7% of CB unstimulated NK cells respectively (n = 4–7). Blockage of TLR2 or TLR4 by neutralizing Abs reduced the proportion of IFN-g producing CD56^bright^ NK cells in response to parasites and IL-15 by 59.7±6.5% and 71.8±8.9% respectively. Simultaneous neutralization of both TLRs did not further inhibit IFN-g release (**Figure 5E**). Altogether, these results indicate that contact-dependent signals between monocytes and CBMC, as well as live parasites and TLR-2 and 4 engagements are needed for NK cell activation.

## Discussion

Our work confirms that *Trypanosoma cruzi* strongly increases the production of IFN-g in response to IL-15 by cord blood NK cells from healthy newborns. The CD56^bright^ NK cell subset is the main responding population. Their activation is associated with up-regulation of surface expression of CD69 and CD54 and down-regulation of CD62L. A low response from CD56^dim^ was also observed. As this NK cell subset outnumbers CD56^bright^ NK cells by meanly 8 fold in peripheral blood [24] its contribution to the final amount of IFN-g detected in supernatants may however be important.

The stimulating action of *T. cruzi* on IL-15-sensitized CB NK cells requires the integrity of parasites as well as contacts between parasites, monocytes and other cells. Moreover, TLR2 and TLR4 engagements and IL-12 produced by monocytes played an important role in the IFN-g response. It is now accepted that NK cells have to be primed by IL-15, after which various other signals delivered by a large variety of receptors may come into play to activate them [31], [32]. In infections, IFN-g production by NK cells can be potentiated by direct contact with pathogens and/or indirectly by cross-talk with myeloid cells that deliver contact-dependant signals and cytokines. Our data support a preferential indirect pathway of NK cell activation by *T. cruzi*, as it is the case in other infections with protozoa like *Leishmania*, *Toxoplasma* and *Plasmodium* [9], [33], [34]. Indeed, TLR2 and TLR4, which are involved in NK cell activation in our conditions and recognize *T. cruzi* molecules [30], [35], were poorly expressed by neonatal NK cells. On the contrary, most monocytes expressed TLR2 and TLR4, sustaining their involvement in an indirect pathway of NK activation by monocytes.

The involvement of monocytes in our system is in line with another recent study underlining the ability of neonatal macrophages to activate NK cells [36]. The role of monocytes could rely to contact-dependent and soluble signals delivered to NK cells. We here show that they synthesize IL-12 and that this cytokine is mandatory for the IFN-g NK cell response to *T. cruzi* and IL-15. It does not exclude the involvement of other signals delivered by monocytes, especially since monocytes also upregulated surface expression of CD40, CD80 and CD83 after *T. cruzi* stimulation (unpublished data) that might also contribute to NK cell activation [9]. IL-12 induction by *T. cruzi* in monocytes is in line with a study of Souza et al. showing the presence of IL-12-positive monocytes in chagasic adult patients [37]. Interestingly, even if we clearly show the induction of IL-12p35 mRNA and of intracellular production of IL-12 by monocytes and the drastic need for IL-12 in our system, we couldn't detect any substantial levels of IL-12 in supernatants by ELISA. This can be due to the fact that too low amounts of IL-12 are present in supernatants to be detected or that IL-12 production is polarized and released in an immunological synapse between monocytes and NK cells in order to be directly used, as reported by Borg et al. [10]. This latter hypothesis is sustained by the observation that contact-dependant signals between monocytes and other cells were needed to induce IFN-g release by neonatal NK cells in response to *T. cruzi* and IL-15. We may however not rule out the existence of additional direct effects of *T. cruzi* on NK cells. Indeed, some pathogens directly drive IFN-g production by NK cells through TLR-2, TLR-4 or other receptors [36], [38], [39]. We showed that these TLRs were expressed by a low proportion of cord blood NK cells and were involved in the NK cell response. Arguing for a potential direct effect of parasites on NK cells, we also observed that *T. cruzi* trypomastigotes induced CD69 expression on purified NK cells (unpublished data).

The fact that mDCs were not involved is quite surprising. Indeed, this cell type is thought to be pivotal for NK cell activation [32] and we recently showed that *T. cruzi* up-regulates the expression, on CB mDCs, of co-activation molecules [40] such as CD80 and CD40, which are able to co-activate NK cells [9]. The differential contribution of monocytes vs. mDCs to activation of CB NK cells may relate to a delayed or insufficient IL-12 production by neonatal mDCs [41], whereas neonatal monocytes might not present same deficiencies to produce IL-12 [42]. Differences in expression of innate receptors might also account for this [43]. Indeed, our data showing that live parasites (able to invade cells) but not lysed ones drive the NK cell response suggest that intracellular receptors need to be engaged in addition to surface TLR2 and TLR4. Human monocytes but not mDCs express the endosomal TLR9, known to recognize *T. cruzi* DNA and to drive IL-12 synthesis [30], [33], [35], [44].

IFN-g production is regulated at multiple transcriptional and post-transcriptional levels [45], [46]. IL-15 drives IFN-g expression by acting mainly at the transcriptional level, triggering the binding of STAT proteins to the regulatory sites of *Ifng* gene promoter [47], [48]. We indeed observed accumulation of IFN-g transcripts in IL-15-primed neonatal NK cells, which was strongly increased when parasites were added. Preliminary studies of actinomycin D chase experiments suggest that the parasite would favor transcription of *Ifng* rather than mRNA stabilization (unpublished data). This is in line with the known ability of IL-12 to induce transcription of *Ifng* [49], the known synergy between IL-12 and IL-15 to induce IFN-g mRNA and protein production by NK cells [25], and the key involvement of IL-12 in the activating effect of *T. cruzi*. The fact that *T. cruzi* would not stabilize IFN-g transcripts is also consistent with the absence of IL-18 production in our conditions, a cytokine shown to increase the half life of IFN-g mRNA [50].

Though substantial, the IFN-g synthesis of IL-15-sensitized CB CD56^bright^ NK cells induced by *T. cruzi* remained slightly inferior to that of adult cells. We observed differences between neonates and adults both at levels of IFN-g transcript accumulation and protein production. We can however notice that the higher response of adult cells seems to preferentially rely on the action of *T. cruzi* rather than on the response to IL-15, which was not that much different between neonates and adults. It is tempting to speculate that the better response of adults reflects differences in accessory cells involved in NK cell activation, such as impaired TLR signalling in early life [41], [51], [52]. Despite of this, it is worth noting that NK cells from all newborns synthesized IFN-g.

As NK cells have to be primed by IL-15 to respond to *T. cruzi*, the source of this cytokine *in vivo* during infection may be questioned. No data are currently available on the ability of *T. cruzi* to induce IL-15 production, except that this cytokine is found *in situ* in hearth tissue from chagasic patients [53]. We could not detect IL-15 in supernatants of CBMC cultured in the presence of trypomastigotes. However, IL-15 is known to be mainly expressed at the surface of myeloid cells, linked to its alpha-chain receptor, which cross-presents it to cells bearing the IL2/IL-15Rβγ [9], [54]. Membrane-bound IL-15 is constitutively produced at low levels in lymph nodes and in the spleen, and our results indicate that the synergistic effect between parasites and IL-15 required only low concentrations of IL-15 that could otherwise not or faintly induce IFN-g. Based on this information, we may raise the following hypothesis about IFN-g NK cell response in congenitally-infected foetuses [17]. *T. cruzi* trypomastigotes pass through placental tissues and directly enters the fetal blood [55]. Blood being filtered by the spleen, parasites might there infect myeloid cells and encounter NK cells, known to be numerous in this secondary lymphoid organ [8], [56]. A mutual cross-talk between parasites, monocytes/macrophages and CD56^bright^ NK cells might therefore occur in the spleen of infected foetuses, where IL-15 is constitutively produced at low levels [57], leading to rapid IFN-g release [12]. Since NK cells can play an important role in induction of primary CD8^+^ T-cell immunity in the absence of CD4^+^ T cells [58], we may presume that the here reported CD56^bright^ NK cell-derived IFN-g is involved *in vivo* to endow myeloid cells to initiate the strong CD8^+^ T cell response observed in newborns congenitally infected with *T. cruzi* [2].

Our work highlights the ability of *T. cruzi* to trigger a robust IFN-g response by IL-15-sensitized NK cells in all neonates, as well as the important role played by IL-12-producing-monocytes, which might partially compensate for the neonatal defects of DCs. Strong activation of NK cells and monocytes may constitutes a way allowing the parasite to overcome the immaturity of the neonatal immune system and favour a type 1 immune response. Our results encourage identifying *T. cruzi* molecules which could play an interesting adjuvant role to improve the efficacy of vaccines, which is necessary to reduce the important morbi-mortality of infectious diseases in early life [5]. They also emphasize the need for complementary studies on CB NK cells activation pathways.
